# Clinical, Etiological, Anatomical, and Bacteriological Study of “Diabetic Foot” Patients: Results of a Single Center Study

**DOI:** 10.7759/cureus.2498

**Published:** 2018-04-18

**Authors:** Ashwinikumar Patil, Datta More, Anant Patil, Kishor A Jadhav, Myriam E Vijil Mejia, Suresh S Patil

**Affiliations:** 1 Neurology, Seth G. S. Medical College and Kem Hospital ,mumbai; 2 More Hospital; 3 Pharmacology, Dr Dy Patil Medical College, Navi Mumbai; 4 Neurology, MGM Hospital, Kamothe, Navi Mumbai; 5 National Autonomous University of Honduras; 6 Sona Hospital, Appasaib Patil Nagar, Sangli, Sona Hospital, Sangli

**Keywords:** clinical assessment, complications, diabetic foot

## Abstract

Objective: To examine the clinical pattern of foot-related complications in type 2 diabetes patients.

Material and methods: A cross-sectional study was conducted among indoor, adult type 2 diabetes patients with risk factors for diabetic foot complications. The diabetic neuropathy symptom score (DNSS), Doppler scanning, ankle brachial pressure index (ABPI) assessment, neuropathy assessment, neuropathic disability score (NDS), biothesiometry evaluation, and bacteriological examination was performed. Diabetic foot risk stratification was done using the NICE risk stratification system. Foot ulcer severity was assessed with the Lipsky severity grading system.

Results: Ninety-one patients (mean age 59 years; male 65.9%) were included, of which 20 (22%) had a history of ulcer and 40 (44%) were smokers. Seventy-seven (83.5%) patients had a neuropathy symptom score between 4 and 9. Biothesiometry vibration perception threshold (VPT) was “severe” in 55 (60.4%) patients. Doppler assessment showed triphasic flow in 53 patients (58.2%). Out of 52 patients (57.1%) with neuropathy, 30 (57.7%) had a severe problem. Diabetic foot ulcer, cellulitis, and callus were present in 44 (48.3%), 29 (31.5%), and 11 (12.4%) patients, respectively. Foot ulcers were present on 21 (38%) metatarsal heads, 11 (20%) toes, 10 (18%) heels, 08 (15%) ankles, and 05 (09%) lateral foot borders. Of the 55 patients who underwent culture examination, 30 (33.3%) showed the presence of Staphylococcus aureus. As per NICE risk stratification, 55 patients (60%) were at “very high risk.”

Conclusion: A foot ulcer is the commonest complication in diabetic patients followed by cellulitis. Standardized simple noninvasive testing methods should be used to identify patients at risk for the diabetic foot. Multidisciplinary diabetic foot care could be useful to prevent diabetes-related amputation of the lower extremities.

## Introduction

Diabetes is a prevalent condition in India. According to the estimates of International Diabetes Federation (IDF 2015), there are over 69 million people with diabetes in India, accounting to a prevalence of 8.7% among people in the age group of 20-79 years [1]. Diabetes is associated with microvascular as well as macrovascular complications in patients with inadequately controlled blood sugar levels. Foot complications in patients with diabetes mellitus are one of the significant medical problems and an economic burden [2]. It also adversely affects the quality of life of patients [3]. An infected foot is one of the important complications in type 2 diabetes mellitus and a risk factor for limb amputation [4]. Ischemia, neuropathy, smoking, duration of diabetes, and inadequate control of diabetes are the risk factors for diabetes-related foot ulcers [5]. Patients may present with ulcers, cellulitis, infections, or a mixed pattern. The prevention of diabetic foot-related complications is important to avoid subsequent problems, including gangrene and amputation. Self-care is a crucial aspect of diabetes management [6]. Identifying risk factors might help to develop better prevention strategies in diabetic patients. The existing foot screening guidelines for diabetes have a significant variability in recommended methods [7]. Similarly, studies related to the pattern of foot-related complications, methods to assess the pattern of presentations, and methods risk stratifications are limited in Indian patients. The present study was performed to examine the clinical pattern of foot-related complications and the associated etiology in patients with type 2 diabetes mellitus.

## Materials and methods

In this cross-sectional descriptive study, adult (>18 years of age) type 2 diabetes indoor patients with risk factors for diabetic foot complications were included. Patients with type 1 diabetes, gestational diabetes mellitus, and human immunodeficiency (HIV) seropositivity/acquired immunodeficiency syndrome (AIDS) were excluded. The study was conducted after taking written, informed consent of the enrolled participants. The diabetic neuropathy symptom score (DNSS) was used for scoring symptoms [7] (maximum score = 9 for each foot, neuropathy (3-9), and vascular (0-3)). A detailed clinical examination was performed to identify the presence of complications related to diabetes. The presence of vasculopathy was checked with Doppler scanning (8 MHz Doppler probe; Handheld 8 MHz Doppler ultrasound - standard pencil style probe (LifeDop, Summit Doppler Systems, Inc., Holland) of the dorsalis pedis artery and the posterior tibial artery. An ankle brachial pressure index (ABPI) assessment was done for all patients. A neuropathy assessment was done using a 10 g monofilament (Semmes-Weinstein, 10 gram/5.07, Diabetic Foot Care India, MES Ltd., Chennai, India), and a 128 MHz tuning fork. The sites of examination for 10 g monofilaments were under the hallux and the first and fifth metatarsals. A 128 MHz tuning fork and hot and cold water test tubes, standard glass test tubes, were applied on the bony part of the dorsal side of the distal phalanx of the first toe). The neuropathic disability score (NDS) was calculated for each patient. Each parameter was tested on the right and left sides and scored as “present” = 0 or “absent.” The maximum total score was 10, categorized into “mild” (3-4), “moderate” (5-6), and “severe” (7-9) [8]. The presence and grade of neuropathy were checked by biothesiometry evaluation (Biothesiometer, i.e., vibrometer Vibration perception threshold (VPT), Diabetic Foot Care India, MES Ltd., Chennai, India) at the distal pulp of the hallux over the bony prominence. A bacteriological examination, or a tissue culture examination, was performed to find out the causative pathogens. Diabetic foot risk stratification was done using the NICE risk stratification system [9]: "low risk": normal sensations, palpable pulses; "at risk": evidence of neuropathy and or absent pulses; "high risk" neuropathy or absent pulses and signs of deformity, skin changes, or previous ulceration; and "very high risk": foot ulcer. Foot ulcer severity was assessed as per the Lipsky severity grading system: “mild” > two signs of inflammation; cellulitis, if present, < 2 cm from the ulcer in the absence of clinical signs of systemic toxicity and infection involving the superficial tissues; “moderate”to “mild’ plus cellulitis > 2 cm from the wound but < 5 cm, no signs of systemic toxicity, and infection spreading to deeper tissue and bone; and “severe”: extensive cellulitis, deep abscess with or without signs of systemic toxicity (fever, vomiting, hypotension, confusion, acidosis, renal failure, severe hyperglycaemia, and leukocytosis). The study was conducted from June 2010 to June 2012.

Categorical data are presented as numbers and percentages whereas continuous data are presented as the mean and standard deviation. Since this was a descriptive study, no inferential tests were applied.

## Results

A total of 91 type 2 diabetes patients (mean age: 59 years) were included in this study. The number and percentages of patients in the age group of 40-50 years, 50-60 years, 60-70 years, and above 70 years were 16 (18%), 35 (38%), 21 (23%), and 19 (21%), respectively. The proportion of male and female patients was 60 (65.9%) and 31 (34.1%), respectively. A total of 20 patients (22%) had a history of previous ulcers, whereas 71 (78%) did not have ulcers in the past. Forty patients (44%) were smokers whereas 51 (56%) were non-smokers (Table 1).

**Table 1:** Baseline characteristics

Parameter	Result
Mean age	59 years
40-50 years n (%)	16 (18%)
51-60 years n (%)	35 (38%)
61-70 years n (%)	21 (23%)
>70 years n (%)	19 (21%)
Male n (%)	60 (65.9%)
Female n (%)	31 (34.1%)
History of previous ulcer n (%)	20 (22%)
History of smoking n (%)	40 (44%)

Seventy-seven patients (83.5%) had a neuropathy symptom score between 4 and 9 (neuropathy range) whereas 14 (16.5%) scored between 0 and 3 (vasculopathy range). The number and percentages of patients with “normal,” “mild,” “moderate,” and “severe.” The neuropathy disability score (NDSs) were 14 (15.7%), 8 (9%), 30 (33.7%), and 39 (41.6%), respectively. The biothesiometry vibration perception threshold (VPT) was in the “severe,” “moderate,” and “mild” ranges in 55 (60.4%), 14 (15.4%), and 08 (8.8%) patients, respectively, whereas 14 patients (15.4%) had “normal” VPT (Table 2).

**Table 2:** Clinical examination findings

Parameter	n (%)
Neuropathy symptom score	
Neuropathy range (4-9)	77 (83.5%)
Vasculopathy range (0-3)	14 (16.5%)
Neuropathy Disability Score (NDS)	
Normal	14 (15.7%)
Mild	08 (08.8%)
Moderate	30 (33.7%)
Severe	39 (41.6%)
Vibration perception threshold (VPT)	
Normal	14 (15.4%)
Mild	08 (08.8%)
Moderate	14 (15.4%)
Severe	55 (60.4%)

A Doppler assessment showed triphasic, biphasic, and monophasic flow in 53 (58.2%), 21 (23.1%), and 17 (18.7%) patients, respectively. A total of 56 patients (61.5%) had ABPI more than 0.9 whereas the remaining 35 (38.5%) had less than 0.9.

**Figure 1:**
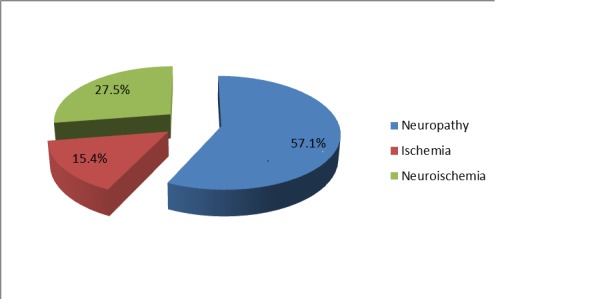
Percentage of patients with signs of neuropathy and ischemia

An etiopathogenesis analysis showed signs of neuropathy in 52 patients (57.1%) whereas ischemia and mixed neuroischemia were seen in 14 (15.4%) and 25 (27.5%) patients, respectively (Figure 1).

**Figure 2:**
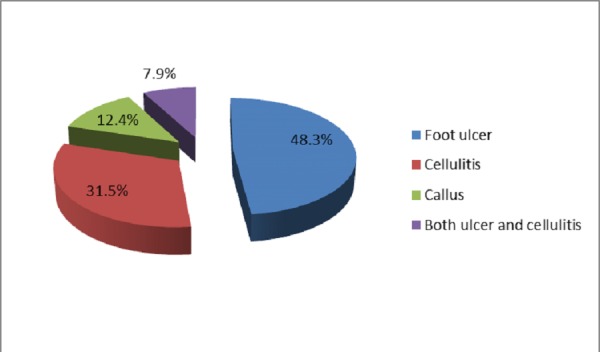
Percentage of patients with diabetic foot complications

Of the 52 patients with neuropathy, 30 (57.7%) had severe neuropathy. Moderate and mild neuropathy was seen in 14 (26.9%) and 08 (15.4%) patients, respectively. Diabetic foot complications in the form of foot ulcer, cellulitis, callus, and ulcer with cellulitis were present in 44 (48.3%), 29 (31.5%), 11 (12.4%), and 07 (7.9%) patients, respectively (Figure 2). 

The locations of the foot ulcer were metatarsal heads: 21 (38%), toe: 11 (20%), heel: 10 (18%), ankle: 08 (15%), and the lateral border of the foot: 05 (09%) (Table 3).

**Table 3:** Location of foot ulcer

Location	n (%)
Metatarsal heads	21 (38%)
Toe	11 (20%)
Heel	10 (18%)
Ankle	08 (15%)
Lateral border of foot	05 (09%)

A total of 55 patients underwent a culture examination. Staphylococcus aureus was isolated in 30 (33.3%) patients, Streptococcus pyogenes in 15 patients (16.7%), and coagulase-negative Staphylococcus in 10 patients (11.1%). The Lipsky severity index was “mild,” “moderate,” and “severe” in 14 (15%), 37 (41%), and 40 (44%) patients, respectively. As per NICE risk stratification, 55 patients (60%) were at “very high risk” whereas 15 (16%), 20 (22%), and 1 (2%) were at “high risk,” “at risk,” and “low risk,” respectively.

## Discussion

In this cross-sectional study, we examined the pattern of foot complications among adult diabetic patients. Age is a known risk factor for neuropathy [10]. Despite our plan to include patients more than 18 years of age, we could not enroll patients below 40 years of age. The mean age for patients with a diabetic foot in another study [11] was 57 years, closely matching our study. The observations suggest that elderly patients should be regularly and carefully evaluated for the risk of diabetic foot. Secondly, diabetic foot syndrome has been reported more commonly in type 2 diabetic males of an older age [12]. In our study, almost two-thirds of the population was male. Diabetes mellitus in male patients has shown to be associated with foot infections and multiple amputations [13] and more likely to lack vibratory perception or posterior tibial pulse and to have calluses [14]. The exact reason for this gender-based difference is unknown.

A past history of ulcer increases the risk of new ulcers in diabetes patients [8]. Similarly, diabetes contributes to a large number of lower extremity amputations and the presence of foot ulcers increases the risk of diabetes-related amputations [15]. In our study, 22% patients had a history of a previous foot ulcer. In another study, 88% of all diabetes-related amputations were preceded by foot ulcers [13]. Smoking is a risk factor for developing diabetic foot complications. A longer duration of diabetes and smoking increases the risk of foot ulcer [15]. The prevalence of smoking in our study was high, indicating its contribution to the development of diabetes-related complications. In diabetes patients with a risk of foot ulcers, a history of ulcers and smoking should not be missed and, if present, patients should accordingly be counseled or suggested measures to prevent complications.

Similar to our observations, symptom score in the neuropathy range has been shown as a common finding [12]. The neuropathy disability score, 10 g monofilament, and palpation of foot pulses are recommended as screening tools in general practice [8]. Neuropathy disability score (NDS) can be used a suitable tool in routine clinical practice to identify patients at risk for developing foot ulcers. 

The tuning fork and monofilament tests, respectively, have lower sensitivity but better specificity and accuracy [16]. In our study, a large number of patients had a biothesiometry vibration perception threshold (VPT) in the severe range. This test can help to follow the patient to examine the course of risk [17]. Age-corrected VPT measurements are objective, simple tests for use in clinical practice and are useful for predicting the risk of foot complications [18]. Ankle reflex is a more sensitive but less specific test [16]. The Biothesiometer and the neuropathy disability score have high sensitivities. The Biothesiometer and the modified neuropathy disability score tend to be more sensitive than the 10 g monofilament for the assessment of risk for foot ulcers. However, some data suggest that the 10 g monofilament may not be the optimum method for identifying patients at risk of foot ulcers [19]. A peripheral vascular disease is a significant predictor of amputation [20], which can be examined by the flow of digital arteries in the lower limbs.

In our study, only 18.7% patients had monophonic flow sounds whereas others had biphasic or triphasic flow sound whereas more than 60% patients had ABPI more than 0.9.

A study reported neuropathic, neuroischemic, and pure ischemic ulcers in 37%, 61%, and 2% patients, respectively. Neuropathy was present in 98% patients with diabetic foot whereas limb ischemia was observed in 63% patients [12]. We also observed neuropathy more commonly than ischemia or mixed pathology, i.e., neuroischemia. However, the number of patients with both neuropathy and ischemia were less in our study. Peripheral neuropathy is prevalent in type 2 diabetic patients, and these patients are far more likely to have complications or co-morbidities [21]. In our study, out of all patients with neuropathy, about 85% had moderate to severe intensity. Foot ulcer and cellulitis were the two most common foot complications in our study patients. Calluses and a combination of both ulcer and cellulitis were also observed in some patients. An ulcer is the most common presentation of diabetic foot [22], whereas plantar callus is a risk factor for ulceration [23]. The great toe is the most common site of callus formation [24]. In our study, metatarsal heads were the most common site of foot ulcer followed by the toe and ankle. Some patients had ulcers on the lateral border of the foot. In another study, 44% foot ulcers were found on the toes and 43% on the plantar surface [12]. Neuropathic causes and the plantar location of ulcer are common in diabetes [25]. Aerobic gram-positive cocci, especially Staphylococci, are the predominant bacteria found in diabetic foot infections [26-27]. In our study too, Staphylococcus aureus was the most commonly isolated pathogen.

The risk assessment and stratification of patients based on the risk could be beneficial in order to individualize treatment. The Lipsky severity index is useful in identifying high-risk patients for lower extremity events [28]. Categorization helps determine the urgency and type of management [26].

High-risk patients need to be advised about foot care, including correcting the shoe that may lead to ulceration [29]. Footwear trauma is a known cause of ulcers [30]. Shoe-foot mismatch should be excluded to reduce the risk of foot ulceration. In addition to these measures, patient education should be continued to reduce the burden of diabetic foot-related complications.

Overall, our study provides many insights regarding foot-related complications in patients with diabetes. However, small sample size and the absence of a control group to examine the comparative pattern are the limitations of our study. Similarly, patients with impaired fasting glucose and impaired glucose tolerance were not included in this study. The duration and compliance of pharmacotherapy could not be confirmed. With these limitations, the observations should be carefully extrapolated.

Further studies with an understanding of risk factors, such as bare feet walking, history of trauma, duration and control of diabetes and hypertension, and an evaluation of outcome will provide more insights about this common complication of diabetes.

## Conclusions

A foot ulcer is the commonest complication in diabetes patients followed by cellulitis. Neuropathy is the most common cause of ulcers in diabetic patients with the common location in the plantar area. Ulcers are typically seen in sites of high mechanical loading due to repetitive trauma in people with loss of pain sensation. Our findings reinforce the importance of using standardized, simple, noninvasive testing methods to identify patients at risk for diabetic foot. Multidisciplinary diabetic foot care including patient education, early detection, effective management of foot problems and scheduled follow-up could be useful to prevent diabetes-related amputation of lower extremities.
